# The Relationship between Dietary Habits and Work Engagement among Female Finnish Municipal Employees

**DOI:** 10.3390/nu14061267

**Published:** 2022-03-17

**Authors:** Jenni Virtanen, Markus A. Penttinen, Marika Laaksonen, Maijaliisa Erkkola, Henna Vepsäläinen, Hannu Kautiainen, Päivi Korhonen

**Affiliations:** 1 Department of General Practice, Turku University and Turku University Hospital, Turku University, 20014 Turku, Finland; mapentti@utu.fi (M.A.P.); paikor@utu.fi (P.K.); 2 Perusturvakuntayhtymä Akseli, 21250 Masku, Finland; 3 Suomen Terveystalo, 20520 Turku, Finland; 4 Fazer Group, 00941 Helsinki, Finland; marika.laaksonen@fazer.com; 5 Department of Food and Nutrition, University of Helsinki, 00014 Helsinki, Finland; maijaliisa.erkkola@helsinki.fi (M.E.); henna.vepsalainen@helsinki.fi (H.V.); 6 Unit of Primary Health Care, Kuopio University Hospital, 70210 Kuopio, Finland; hannu.kautiainen@medcare.fi; 7 Folkhälsan Research Centre, University of Helsinki, 00290 Helsinki, Finland

**Keywords:** work engagement, nutrition, mental health

## Abstract

Background: Work engagement reflects work-related well-being. It is positively associated with health, life satisfaction, work efficiency, income level, and occupational prospects. However, little is known about the relationship between work engagement and diet. Methods: A cross-sectional study was conducted among female Finnish municipal employees (*n* = 630) in 2015. Work engagement was assessed using the Utrecht Work Engagement Index. The consumption of healthy and unhealthy food items was determined using a food frequency questionnaire. Sociodemographic factors, health behaviors, depressive and anxiety symptoms were assessed with self-administrated questionnaires. Results: Work engagement had a positive relationship with the daily consumption of healthy food items. This association remained significant even after adjusting for age, education years, financial situation, and physical activity. The frequency of consuming unhealthy food items showed no relationship with work engagement. Anxiety and depressive symptoms decreased linearly with the greater consumption of healthy foods. Conclusion: Frequent consumption of healthy food items is associated with higher work engagement, irrespectively of the consumption of unhealthy nutrients. These results encourage health care professionals to recommend healthy food items instead of forbidding unhealthy food, as well as employers to support healthy dietary habits among employees.

## 1. Introduction

Work engagement is a positive psychological construct which is defined as “a positive, fulfilling, work-related state of mind that is characterized by vigor, dedication, and absorption” [1]. Rather than a momentary and specific state, engagement refers to a more persistent and pervasive affective-cognitive state that is not focused on any particular object, event, individual, or behavior [1]. High work engagement has been related to not only better work efficiency, but also to better health and higher life satisfaction [1,2]. In contrast, low work engagement has been associated with burnout, depressive symptoms and other psychosocial risk factors [1,3,4,5]. In a recent study among Finnish employees, Hakanen et al. indicated that high work engagement predicted better income levels and was positively related to occupational prospects. High work engagement also protected against future unemployment and disability pensions [6].

Previous studies about work engagement have mostly focused on social and organizational aspects. To the best of our knowledge, there only two previous studies have investigated the association between employee’s lifestyle behaviors and level of work engagement [7,8]. Both of these studies suggested that healthy lifestyle choices (e.g., physical exercise and healthy diet) were positively associated with work engagement [7,8].

The importance of a healthy diet (e.g., Mediterranean or Nordic diet) preventing the development of several chronic diseases has been established [9,10,11,12]. Moreover, a Mediterranean diet may have a role in preventing cognitive impairment and dementia, although the evidence is not unambiguous [13]. Furthermore, in a randomized controlled trial, Nilsson et al. showed that a balanced diet might even improve cognitive performance in apparently healthy adults [14]. The biological mechanism is indefinite, but studies suggest that an anti-inflammatory diet plays a major role in enhancing cognition, and in the prevention of mental disorders such as depression [14,15].

Previous research by our study group indicated that a high consumption of healthy food items was associated with lower levels of burnout symptoms [16]. Considering the relationship between burnout and work engagement [1], these observations prompted us to more specifically investigate how various food items are associated with work engagement. Our research question was: “Is consumption rate of healthy food items associated with work engagement among female employees?” We also hypothesized that unhealthy food items are negatively associated with work engagement.

## 2. Material and Methods

### 2.1. Participants

This cross-sectional study was a part of the PORTAAT-study (Pori To Aid Against Threats) conducted among municipal employees of the city of Pori (83,497 inhabitants in 2014) in southwestern Finland in 2014–2015. Information and invitation letters to participate in the study were sent as an email attachment to the employees (*n* = 2570) by the managers of the ten selected work units. The response rate was 33%; in total, 836 employees (104 men, 732 women) consented to participate in the study There were representatives from many different professions participating in the study (e.g., librarians, cleaners, museum employees, groundkeepers, kitchen staff, computer workers, social workers, nurses, physicians, administrative officials, and general office staff). The present analyses are restricted only to female subjects, because there were so few male participants. For the present study, we reported the data of the 630 female participant who attended the follow up in the year 2015.

### 2.2. Physical Examination

Physical examinations were performed by trained study nurses. Blood pressure was measured with an automatic validated blood pressure monitor, with subjects in a sitting posture after resting for at least 5 min. Two readings taken at intervals of at least 2 min were measured, and the mean of these readings was used in the analysis. Height and weight were measured with subjects in a standing position without outer garments and shoes. Weight was measured to the nearest 0.1 kg with calibrated scales and height to the nearest 0.5 cm with a wall-mounted stadiometer. Body mass index (BMI) was calculated as the weight (kg) divided by the square of the height (m^2^). Waist circumference was measured with the participant in a standing position at a level midway between the lower rib margin and the iliac crest.

Information about regular medication and previously diagnosed diseases were collected from the self-administrated questionnaires and medical records. Laboratory tests were determined on blood samples which were obtained after at least 8 h of fasting. Plasma glucose, total cholesterol, high-density lipoprotein cholesterol (HDL-C), and triglycerides were measured enzymatically (Architect c4000/c8000). Low-density lipoprotein cholesterol (LDL-C) was calculated with the Friedewald formula.

### 2.3. Work-Related Factors

The type of duty (daytime or shift work) and the average working hours per week were obtained from the self-administrated questionnaires.

Work engagement was evaluated with the Utrecht Work Engagement Index (UWES-9), which has been validated in a Finnish population [17]. In these previous analyses, UWES-9 scores were categorized as <1.44 (very low), 1.44–3.43 (low), 3.44–4.53 (moderate), 4.54–5.30 (high) and 5.31–6.00 (very high) [17]. UWES-9 consists of three subscales—vigor, dedication and absorption—and each subscale consists of three items. The items for vigor are: “At my work, I feel bursting with energy”, “At my job, I feel strong and vigorous” and “When I get up in the morning, I feel like going to work”. For dedication: “I am enthusiastic about my job”, “I am proud on the work that I do” and “My job inspires me”. For absorption the items are: “I am immersed in my work”, “I get carried away when I’m working” and “I feel happy when I am working intensely”. The items are scored on a 7-point Likert scale, ranging from 0 (never) to 6 (daily) [1,18,19]. The mean subscale score was computed by adding the scores on the particular scale and dividing the sum by the number of items in the subscale involved. A similar procedure was followed for the total score. The higher each item was rated, the higher the overall work engagement.

### 2.4. Health Behavior and Other Measures

Physical activity (PA) was determined with a self-administrated questionnaire with the duration and frequency of leisure time and commuting activities in a typical week. For the present analyses, moderate aerobic-type PA (e.g., brisk walking) and vigorous aerobic-type PA (e.g., running) were taken into account. Alcohol consumption was assessed using the 3-item Alcohol Use Disorders Identification Test (AUDIT-C), with a cut-off of 5 points for harmful alcohol use in women [20,21].

Smoking status was assessed with a questionnaire. Non-smoking was defined as having never smoked or having quit smoking >12 months ago.

Information about the marital status (cohabiting or not), number of years of education (starting from primary school), financial satisfaction (with the question “I have to spare in my expenditures”, yes or no) and sleep quality (good or poor) were collected from self-administrated questionnaires.

### 2.5. Psychosocial Measures

The Major Depression Inventory (MDI) was used to evaluate depressive symptoms. MDI is a self-rated questionnaire consisting of ten items, of which two have a sub-item (twelve items altogether), of which only the higher is included in the total score. MDI measures depressive symptoms during the past two weeks on a 6-point Likert-type scale, from 0 = never to 5 = all the time. Total score ranges from 0 to 50, with a higher score indicating more depressive symptoms [22].

Anxiety symptoms were assessed with the General Anxiety Disorder 7-item Scale (GAD-7). The total score is from 0 to 21; 0–4 = no or little anxiety, 5–9 = some anxiety, 10–15 = substantial anxiety and 16–21 = severe anxiety; a score of 10 or more has a sensitivity of 89% and a specificity of 82% for generalized anxiety [23].

### 2.6. Food Frequency Questionnaire

The subjects reported their food consumption frequencies during the past week using a 45-item food frequency questionnaire (FFQ). The FFQ was based on the version developed for and validated among children [24], with specific attention paid to capture the consumption patterns of vegetables and fruits, as well as sugary foods and beverages. A shortened, electronic, 25-item version of the FFQ has been tested for reproducibility with mostly moderate (0.40–0.60) or good (0.61–0.80) intraclass correlation coefficients [25]. The FFQ used in the present study included eight food groups: fruits, vegetables and berries; dairy products; fats and oils; fish; meat and eggs; cereal products; drinks; and others (i.e., sweets and snacks). The frequencies of meals (breakfast, lunch, dinner and snacks) and the use of dietary supplements were included. The FFQ had three answer columns: not at all, times per week and times per day. The instruction was to either tick the “not at all” column or to write a number in one of the other columns.

The main food groups in the FFQ were categorized into healthy and unhealthy food items (Table 1 based on previous scientific understandings of the association between diet and cognitive health [14,26] and the Nordic Nutrition Recommendations for a healthy and balanced diet [27]. From the data collected with the FFQ, we summarized the mean daily consumption frequency of food items in the same group, and used that as an indicator of dietary consumption in the statistical analysis studying the association between diet and work engagement. For example, the daily consumption frequency of whole grain breads, pasta, rice, porridge, muesli, etc., was summarized and used as an indicator of whole-grain product consumption. We did not have the data on energy intake; therefore, we adjusted the statistical analysis with physical activity to include the effects of the energy intake in the model.

### 2.7. Statistical Analyses

Data are presented as means with standard deviation (SD) or as counts (*n*) with percentages (%). The consumption of healthy and unhealthy food items (per day) was divided into four categories containing 20%, 30%, 30%, and 20% (20th, 50th, and 80th percentiles) of the total distribution. The unadjusted hypothesis of linearity was tested using the Cochran–Armitage test, analysis of variance or Poisson regression models with an appropriate contrast. The adjusted hypothesis of linearity (orthogonal polynomial) was evaluated using generalized linear models (e.g., analysis of covariance and Poisson regression models) with appropriate distribution and link function. Models included age, education years, financial situation, and PA hours per week as covariates when appropriate. A possible nonlinear relationship between total work engagement score and the consumption of healthy and unhealthy food items was assessed using a 4-knot-restricted cubic spline regression model. Knot locations were based on Harrell’s recommended percentiles [28]. Food item consumption was skewed; therefore, they were transformed to normality using van der Waerden’s rank-based normalization methods [29]. Normal distributions were evaluated graphically and with the Shapiro–Wilk test. The Stata 16.1 (StataCorp LP; College Station, TX, USA) statistical package was used for analyses.

## 3. Results

We evaluated 630 female participants with a mean age of 49 (SD 10) years. Table 2 shows the characteristics of the subjects according to categories of healthy food items consumed per day. Participants with a higher intake of healthy food items were older, more satisfied with their financial situation, were more physically active, ate breakfast more regularly, and had a lower alcohol consumption and lower prevalence of smoking than women consuming less healthy food items. Systolic blood pressure was positively associated with the increased consumption of healthy food items, whereas plasma triglyceride and glucose levels were negatively associated with the increased consumption of healthy food items. The mean MDI and GAD-7 scores were lowest among subjects with the highest intake of healthy food items.

Table 3 shows the healthy and unhealthy food items and average consumption rates per day in the categories of healthy food item usage. Subjects who consumed healthy food items more frequently consumed them from all healthy food item categories, but there was no statistically significant linearity in the consumption of unhealthy food items. The consumption of red meat, sugar-sweetened juices and beverages and sweet bakery products was more frequent among those who otherwise ate in a healthier way.

**Figure 1:**
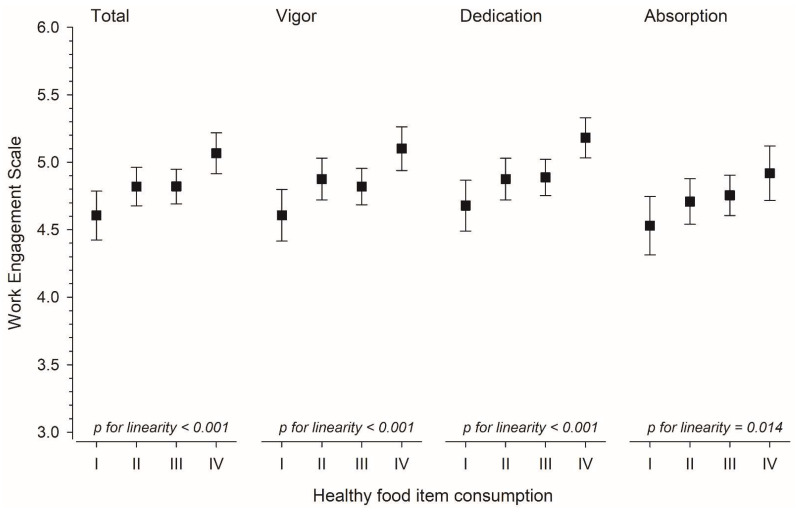
Mean with 95% confidence intervals of total work engagement and its subscales (adjusted for age, education years, financial situation, and physical activity hours per week) according to the categories of healthy food items consumed per day.

Figure 1 shows that total work engagement and its components had a positive relationship with healthy food item consumption per day when adjusted for age, years of education, financial situation, and physical activity hours per week.

**Figure 2:**
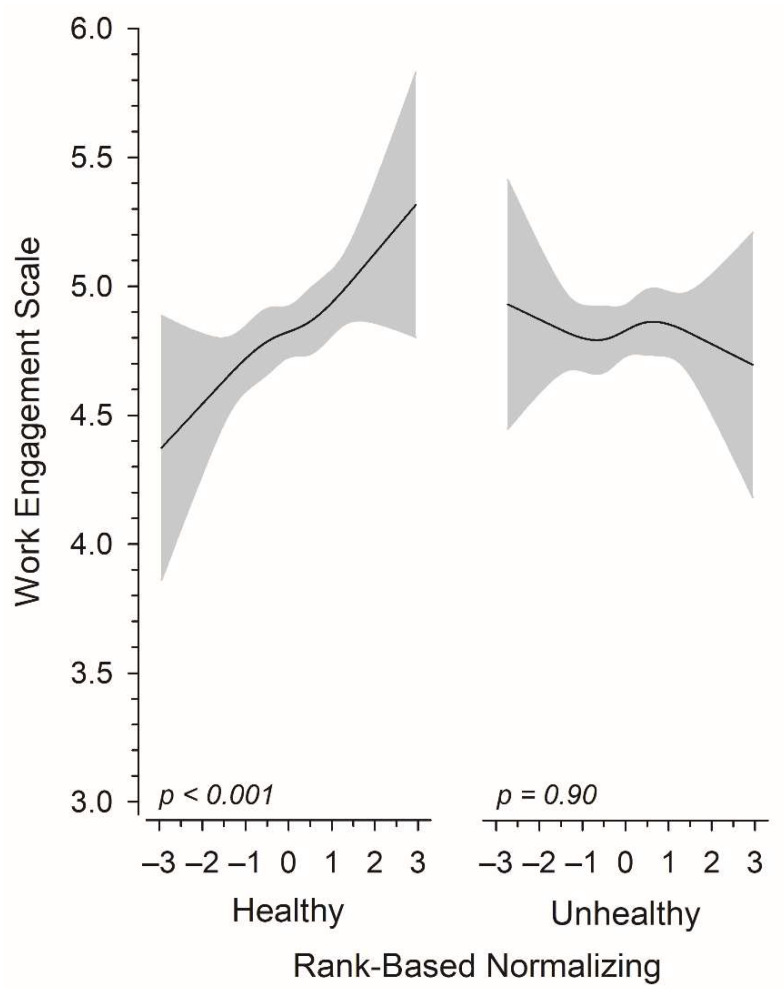
Relationships of total work engagement score as a function of the rank-based normalizing level of healthy and unhealthy food item consumption. The curves were derived from a 4-knot-restricted cubic splines regression models. The models were adjusted for age, education years, financial situation, physical activity hours per week, and the number of the unhealthy or healthy food items, respectively. The gray area represents the 95% confidence intervals.

Figure 2 illustrates the continuous relationship between total work engagement and standardized score (z score) for the consumption of healthy and unhealthy food items. Adjustments were made for age, years of education, financial situation, physical activity hours per week, healthy food items with unhealthy items and vice versa. Subjects consuming healthy food items more frequently per day had higher work engagement, whereas the consumption frequency of unhealthy food items showed no relationship with the total work engagement score.

## 4. Discussion

Our results suggest that the frequent consumption of healthy food items is associated with higher work engagement, irrespectively of the consumption of unhealthy nutrients. The association was observed in all three subscales of work engagement. Interestingly, there was no association between the consumption of unhealthy food items and work engagement.

The results are in agreement with the few previous studies indicating the association between work engagement and dietary choices [7,8]. In a cross-sectional study conducted among Japanese employees, a bi-directional pathway was suggested, i.e., higher work engagement promotes healthier lifestyle habits, and vice versa. It was also speculated that by improving employees’ work engagement, it might be possible to positively impact peoples’ dietary habits [8].

One possible mechanism by which a healthy diet may directly improve mood and cognitive capacity is to influence the inflammatory status of the body. Low-grade inflammation has been linked with the generation of several diseases, including cardiovascular disease and depression [30,31]. There is evidence that a balanced and healthy diet decreases low-grade inflammation and may thereby influence not only cardiometabolic risk factors, but also cognitive capability [13,14,32].

On the other hand, it has been postulated that an unhealthy diet may contribute to low-grade systemic inflammation, with subsequent psychiatric disorders such as depression [15,30,33]. In the present study, depressive and anxiety symptoms were more prevalent among those who ate healthy food items less frequently (Table 2). However, there was no negative association between the consumption of unhealthy (and proinflammatory) diet and low work engagement. In fact, the consumption of certain unhealthy and proinflammatory food items (red meat, juices and beverages sweetened with sugar, and sweet bakery products) was more frequent among those who otherwise ate in a healthier way. One possible explanation for this finding is that the high consumption of healthy anti-inflammatory food items is sufficient to inhibit harmful low-grade inflammation in the body induced by proinflammatory food items. Further studies are required to address this question.

Another possible explanation for the association between high work engagement and healthy diet is that the diet can directly influence gut microbiota [34]. Recent studies suggest that the gut microbiota may play a significant role in the generation of mental diseases such as depression [35]. This occurs, at least partly, through the gut–brain axis, which has been extensively studied in recent years. The gut–brain axis is a bidirectional communication between the central and the enteric nervous system, linking the emotional and cognitive centers of the brain to the peripheral intestinal functions [36]. Further research with a longitudinal study design is needed to determine whether work engagement can be improved by influencing the gut microbiota with a healthier diet.

It is known that healthy lifestyle choices tend to accumulate. This phenomenon was also observed in our study. As demonstrated in Table 2, the subjects who ate more healthy food items had also healthier lifestyle habits. Moreover, the participants who ate healthy food items more frequently consumed more from all healthy food item categories (Table 3). They also had a regular daily breakfast more often than participants with unhealthier dietary choices. The importance of a regular breakfast has been established in weight control [37]. In contrast to our expectations, there were no statistically significant differences between the groups in the clinical measures of overweight/obesity.

### Strengths and Limitations of the Study

Notably, the FFQ designed for this study only assessed the frequency, not the quantity, of the food items. Therefore, a notable limitation of the FFQ is that portion sizes were not determined; thus, the total amounts of food consumed cannot be determined.

The major limitation of our study is the cross-sectional design. Thus, we cannot confirm the causality of healthy food items with work engagement. In addition, dietary habits and other lifestyle behaviors were measured by self-assessment, which increases the risk of inaccurate reporting.

We investigated a female population as being part of an active workforce, which limits the generalizability of the results. However, our study population is quite representative of female employees working in the public sector in Nordic countries. The study population consisted of participants from many different professions, from computer workers to nurses, whose work environments vary from light office work to physically challenging nursing work. Our study population was quite affluent, because individuals outside the workforce were not included. This also limits the generalizability of the results

In addition, many aspects of life were taken into account, and clinical examinations were performed by trained study nurses. The participation rate of 33% was not good, but it is known that email surveys have about a 20% lower response rate than mail surveys [38].

## 5. Conclusions

In conclusion, the frequent consumption of healthy food items was positively associated with work engagement in municipal female employees, whereas consuming unhealthy food items had no relationship with work well-being as assessed by work engagement. Further research is needed to determine whether work engagement can be improved by consuming a healthy diet. The results suggest that employers would be wise to support employees’ healthy dietary habits and provide healthy lunch options, for example, which might even improve work efficiency. Our results also encourage physicians to recommend healthy food items instead of forbidding unhealthy food when giving dietary counseling to patients.

## Figures and Tables

**Table 1:** Food groups considered healthy or unhealthy.

Healthy Food Groups	Unhealthy Food Groups
Fat free milk and sour milk, low-fat cheese (fat <20%)	Red meat, sausages, red cold meat
Unflavoured nuts, seeds and almonds	Juices and beverages sweetened with sugar
Legumes (peas, lentils, beans)	Savoury bakery products such as pies and pastries, potato chips and nachos, popcorn, salty nuts
Fresh vegetables	Sweet bakery products (buns, pies, cookies, cakes), chocolate, sweets
Fresh fruits and berries	Alcohol
Whole grain pasta and rice, rye bread, rye crisp bread, breakfast cereal, muesli, porridge	High-fat dairy products: full fat milk and sour milk, full-fat cheese (fat >20%), butter, butter-oil spreads (fat >80%)
Fish and fish dishes	
Margarines and oils (cooking, bread spread, salad dressing)	
Cooked vegetables	
Eggs	
White meat	

**Table 2:** Characteristics of the study subjects according to categories of healthy food items consumed per day.

	Categories of Healthy Food Items Consumed per Day *				*p* for Linearity
	I *n* = 126	II *n* = 189	III *n* = 189	IV *n* = 126	
Sociodemographic factors					
Age, years, mean (SD)	48 (10)	47 (9)	49 (10)	52 (9)	0.002
Education years, mean (SD)	13.7 (2.7)	14.1 (2.6)	14.0 (2.8)	14.1 (2.7)	0.52
Cohabiting, *n* (%)	94 (75)	149 (79)	156 (83)	106 (84)	0.036
Financial satisfaction, *n* (%)	78 (62)	135 (71)	144 (76)	99 (79)	0.002
Working hours, hours/week, mean (SD)	36.0 (10.9)	36.0 (10.0)	36.4 (5.9)	37.0 (9.7)	0.33
Shift work, *n* (%)	38 (30)	61 (32)	44 (23)	29 (23)	0.055
Health behaviors					
PA, hours per week, mean (SD)	2.0 (3.5)	2.5 (2.2)	2.8 (3.2)	2.9 (2.2)	0.004
Good quality of sleep, *n* (%)	87 (69)	157 (83)	139 (74)	96 (76)	0.68
Daily breakfast, *n* (%)	98 (78)	173 (92)	176 (93)	115 (91)	<0.001
AUDIT-C, mean (SD)	3.0 (1.6)	2.7 (1.6)	2.7 (1.7)	2.7 (1.5)	0.11
Current smoking, *n* (%)	19 (15)	15 (8)	10 (5)	8 (7)	0.008
Clinical factors					
Major Depression Inventory, mean (SD)	6.5 (6.3)	4.2 (4.9)	6.1 (6.7)	3.7 (4.1)	0.018
General Anxiety Scale, mean (SD)	3.4 (3.3)	2.7 (3.2)	3.3 (3.7)	2.1 (2.7)	0.028
Blood pressure, mmHg, mean (SD)					
Systolic	129 (16)	130 (17)	132 (18)	133 (18)	0.029
Diastolic	85 (9)	84 (11)	85 (10)	84 (11)	0.95
Height, cm, mean (SD)	164 (6)	165 (6)	166 (6)	165 (6)	0.089
Weight, kg, mean (SD)	72.7 (15.7)	74.2 (15.7)	72.7 (13.2)	71.7 (12.2)	0.36
Body mass index, kg/m^2^, mean (SD)	26.9 (5.3)	27.2 (5.2)	26.6 (4.6)	26.2 (4.3)	0.11
Waist, cm, mean (SD)	90 (14)	90 (13)	88 (12)	88 (12)	0.081
Total cholesterol, mmol/L, mean (SD)	5.40 (0.99)	5.22 (0.85)	5.16 (0.94)	5.36 (0.88)	0.58
LDL cholesterol, mmol/L, mean (SD)	3.11 (0.78)	2.98 (0.71)	2.89 (0.77)	3.03 (0.69)	0.24
HDL cholesterol, mmol/L, mean (SD)	1.76 (0.42)	1.76 (0.43)	1.80 (0.45)	1.84 (0.48)	0.11
Triglycerides, mmol/L, mean (SD)	1.20 (0.57)	1.09 (0.57)	1.06 (0.56)	1.08 (0.59)	0.12
Fasting glucose, mmol/L, mean (SD)	5.60 (0.56)	5.45 (0.46)	5.51 (0.64)	5.43 (0.50)	0.041
Antihypertensive medication, *n* (%)	24 (19)	28 (15)	39 (21)	19 (15)	0.84
Antilipidemic medication, *n* (%)	3 (2)	5 (3)	12 (6)	10 (8)	0.011

Abbreviations: AUDIT-C, Alcohol Use Disorders Identification Test; LDL, low-density lipoprotein; HDL, high-density lipoprotein; PA, physical activity; SD, standard deviation. * The consumption of healthy and unhealthy food items (per day) was divided into four categories containing 20%, 30%, 30%, 20% (20th, 50th, and 80th percentiles) of the total distribution. Category I: Participants consuming healthy food items fewer than 7.5 times per day. Category II: Participants consuming healthy food items 7.5–10.4 times per day. Category III: Participants consuming healthy food items 10.5–14.7 times per day. Category IV: Participants consuming healthy food items more than 14.7 times per day.

**Table 3:** Average consumption of healthy and unhealthy food items according to the consumption of healthy food items consumed per day.

	Categories of Healthy Food Items Consumed per Day *				*p* for Linearity
	I *n* = 126 Mean (SE)	II *n* = 189 Mean (SE)	III *n* = 189 Mean (SE)	IV *n* = 126 Mean (SE)	
Total consumption of healthy food items	6.02 (0.11)	9.04 (0.06)	12.50 (0.08)	17.96 (0.26)	<0.001
Fat-free milk and sour milk, low-fat cheese (fat <20%)	0.46 (0.05)	0.91 (0.06)	1.70 (0.09)	2.73 (0.17)	<0.001
Unflavored nuts, seeds and almonds	0.15 (0.02)	0.28 (0.03)	0.39 (0.03)	0.72 (0.08)	<0.001
Legumes	0.05 (0.01)	0.15 (0.02)	0.23 (0.03)	0.23 (0.03)	<0.001
Fresh vegetables	0.93 (0.04)	1.28 (0.04)	1.63 (0.06)	2.26 (0.09)	<0.001
Fruits and berries	1.12 (0.05)	1.57 (0.04)	2.10 (0.07)	2.90 (0.12)	<0.001
Whole grain products	1.35 (0.07)	1.93 (0.06)	2.54 (0.08)	3.49 (0.14)	<0.001
Fish and fish dishes	0.23 (0.01)	0.25 (0.01)	0.31 (0.02)	0.41 (0.03)	<0.001
Margarine and oils	0.75 (0.06)	1.33 (0.06)	2.01 (0.08)	3.12 (0.13)	<0.001
Cooked vegetables	0.48 (0.03)	0.69 (0.03)	0.82 (0.04)	1.07 (0.06)	<0.001
Eggs	0.24 (0.02)	0.29 (0.02)	0.37 (0.03)	0.43 (0.04)	<0.001
White meat	0.25 (0.02)	0.34 (0.02)	0.43 (0.03)	0.60 (0.06)	<0.001
Total consumption of unhealthy food items	3.93 (0.20)	3.82 (0.14)	3.92 (0.16)	4.28 (0.24)	0.057
Red meat, sausages, red cold meat	1.09 (0.07)	1.15 (0.06)	1.33 (0.06)	1.39 (0.09)	<0.001
Juices and beverages sweetened with sugar	0.18 (0.04)	0.12 (0.02)	0.09 (0.02)	0.07 (0.02)	0.021
Savory bakery products	0.16 (0.02)	0.15 (0.02)	0.16 (0.02)	0.19 (0.02)	0.10
Sweet bakery products	0.87 (0.06)	0.96 (0.05)	1.02 (0.07)	1.15 (0.09)	0.003
Alcohol	0.16 (0.02)	0.14 (0.01)	0.15 (0.02)	0.19 (0.03)	0.32
High-fat dairy products	1.47 (0.12)	1.30 (0.08)	1.17 (0.09)	1.29 (0.13)	0.34

* The consumption of healthy and unhealthy food items (per day) was divided into four categories containing 20%, 30%, 30%, 20% (20th, 50th, and 80th percentiles) of the total distribution. Category I: Participants consuming healthy food items less than 7.5 times per day. Category II: Participants consuming healthy food items 7.5–10.4 times per day. Category III: Participants consuming healthy food items 10.5–14.7 times per day. Category IV: Participants consuming healthy food items more than 14.7 times per day.

## Data Availability

The datasets used and/or analyzed during the current study are available from the corresponding author on request.
